# Otoacoustic Estimate of Astronauts’ Intracranial Pressure Changes During Spaceflight

**DOI:** 10.1007/s10162-024-00962-1

**Published:** 2024-09-13

**Authors:** Arturo Moleti, Triestino Minniti, Yoshita Sharma, Altea Russo, Andrea Civiero, Maria Patrizia Orlando, Robert MacGregor, Marco Lucertini, Arnaldo D’Amico, Giorgio Pennazza, Marco Santonico, Alessandro Zompanti, Alessandro Crisafi, Maurizio Deffacis, Rosa Sapone, Gabriele Mascetti, Monia Vadrucci, Giovanni Valentini, Dario Castagnolo, Teresa Botti, Luigi Cerini, Filippo Sanjust, Renata Sisto

**Affiliations:** 1 Department of Physics, University of Rome Tor Vergata, Rome, Italy; 2 University of Trieste, Trieste, Italy; 3 CNR-IMM, Sez. Acustica e Sensoristica, Rome, Italy; 4 Airbus US Space and Defense, Inc. and, NASA Johnson Space Center , Houston, TX USA; 5 Italian Air Force–Aerospace Medicine Dept., Experimental Aerospace Division, Rome, Italy; 6 Department of Electronic Engineering, University of Rome Tor Vergata, Rome, Italy; 7 University Campus Bio-Medico, Rome, Italy; 8 ALTEC Spa, Turin, Italy; 9 ASI-Italian Space Agency, Rome, Italy; 10 Telespazio, Naples, Italy; 11 INAIL Research, DIMEILA, Monte Porzio Catone (Rome), Italy

**Keywords:** Microgravity, Otoacoustic emissions, Middle ear transmission, Astronaut physiology

## Abstract

**Purpose:**

To investigate the potential correlation between prolonged exposure to microgravity on the International Space Station and increased intracranial fluid pressure, which is considered a risk factor for the astronauts’ vision, and to explore the feasibility of using distortion product otoacoustic emissions as a non-invasive in-flight monitor for intracranial pressure changes.

**Methods:**

Distortion product otoacoustic emission phase measurements were taken from both ears of five astronauts pre-flight, in-flight, and post-flight. These measurements served as indirect indicators of intracranial pressure changes, given their high sensitivity to middle ear transmission alterations. The baseline pre-flight ground measurements were taken in the seated upright position.

**Results:**

In-flight measurements revealed a significant systematic increase in otoacoustic phase, indicating elevated intracranial pressure during spaceflight compared to seated upright pre-flight ground baseline. Noteworthy, in two astronauts, strong agreement was also observed between the time course of the phase changes measured in the two ears during and after the mission. Reproducibility and stability of the probe placement in the ear canal were recognized as a critical issue.

**Conclusions:**

The study suggests that distortion product otoacoustic emissions hold promise as a non-invasive tool for monitoring intracranial pressure changes in astronauts during space missions. Pre-flight measurements in different body postures and probe fitting strategies based on the individual ear morphology are needed to validate and refine this approach.

## Introduction

The hypothesized intracranial pressure (ICP) increase in prolonged microgravity conditions is considered one of the main potential risks for the astronauts’ health. The risk of damage to the retina and to the optical nerve [1–3] is related to the development of the spaceflight-associated neuro-ocular syndrome (SANS), previously known as visual impairment and intracranial pressure (VIIP) syndrome. Lumbar puncture (LP) taken 12 to 60 days after return to Earth in four ISS astronauts [1, 2] demonstrated elevated LP opening pressure values (from 15.4 to 20.6 mmHg), but the ICP increase during spaceflight has not been demonstrated yet.

As the direct LP measure of ICP is an invasive method, reliable non-invasive indirect approaches are necessary to measure ICP on the International Space Station (ISS). One of the most promising methods is based on the variation of the otoacoustic emission (OAE) phase. The cochlear lymphatic fluids are in contact with the cerebrospinal fluids, so the increase of the pressure in the peripheral ear is a good proxy for the pressure of the intracranial fluid [4]. The increased pressure causes an increase of the middle ear reflectance [5], and as a direct consequence, the OAE phase increases. Avan et al. [6] presented a model in which the increased stiffness of the middle ear is parametrized by the decrease of the capacitive component of the impedance, mainly in the low frequency range.

In the present study, we will use OAE phase measurements to detect the astronauts’ ICP changes in microgravity during long-term missions on the ISS.

### ICP and Otoacoustic Emission Phase and Level

A reliable quantitative relation between the OAE phase variation and the ICP change comes from studies on pathological subjects in which controlled ICP was induced and invasively measured for ethically justified medical purposes. A linear relation between the OAE phase and the ICP variations has been proposed by Avan et al. [7], based on data by Büki et al. [8], obtained during neurosurgery:

1 $$\frac{\partial \varPhi }{\partial \text{ICP}}=2.72 \ \text{deg}/\text{mmHg}$$

The results by Williams et al. [9] about the relation between DPOAE phase change and induced controlled ICP increase agree roughly with Eq. (1), suggesting also that the low-frequency (about 1 kHz) DPOAE phase may be relatively more sensitive to ICP changes. This observation agrees with the results obtained by Bershad et al. [10], who measured ICP and DPOAE changes (magnitude and phase) in 20 patients undergoing lumbar puncture. The OAE level change measurements require specifying the acquisition method and the stimulus calibration (if any) adopted in the study, because they are obviously sensitive to the actual stimulus level reaching the cochlea. The OAE level may change, as the phase, due to changes in both forward and backward middle ear transmission, but it could also be affected by changes in the working point or in the effectiveness of the cochlear amplifier.

### ICP Changes Induced by Postural Changes

The present study compares DPOAE phase measured in 0G on the ISS in a straight relaxed body posture to those measured pre-flight in the seated upright position. As most 0G ICP studies use a different baseline position (supine), it is necessary to discuss the effect of body posture on the ICP measurements, both direct and OAE-based. In free-falling reference frames as the ISS or an aircraft during parabolic flight, there is no preferred direction to define a horizontal or vertical body posture, but the curled or straight body posture could still make a difference.

In terrestrial gravity (1G), ICP is sensitive to the subject’s body posture, as demonstrated by several experiments, summarized in Table 1. Curled or straight body positions also yield different ICP values [11]. In the horizontal position, flexing the hips did not systematically change ICP, while flexing the neck increased ICP by 3.7 (with straightened hips) to 5.5 mmHg (with flexed hips). Focusing on the difference between seated and supine position, direct ICP measurements [11–14] found differences between 10 and 16 mmHg between seated and supine position.

**Table 1:** Postural 1G experiments

Avan [7]	DPOAE	40 subjects	Supine 1G–seated 1G: + 15° (+ 6 mmHg)
Sisto [15]	DPOAE	15 subjects	Supine 1G–seated 1G: + 16° (+ 6 mmHg)
Jasien [16]	TEOAE	Right ear of 13 astronauts	Supine 1G–seated 1G: + 17° (+ 6.5 mmHg) –15° HDT–seated 1G; + 39° (+ 15 mmHg)
Lawley [14]	ICP (Ommaya reservoir)	8 subjects	Supine 1G–seated 1G: + 11 mmHg
Pedersen [11]	ICP (LP)	Vertical: 24 subjects Horizontal: 15 subjects	–4.3 mmHg (sitting upright) 11.9 mmHg (supine) 13 mmHg (vertical with neck flexion) –2.3 mmHg (vertical with neutral neck) 20.5 mmHg (horizontal-neck and hip flexion) 18.7 mmHg (horizontal-neutral hip) 14 mmHg (horizontal-neutral neck) 15 mmHg (horizontal-neutral neck and hip)
Petersen [12]	ICP (LP)	9 subjects	–20°: 20 ± 4.7 mmHg 0°: 8.9 ± 3.7 mmHg 90°: –5.4 ± 5 mmHg
Eklund [13]	ICP (LP)	11 subjects	10.5 ± 1.5 mmHg (first supine) –0.8 ± 3.8 mmHg (sitting) 11.5 ± 0.8 mmHg (second supine) 15.8 ± 1.3 mmHg (head down tilt)

ICP changes associated with postural changes in 1G have been detected also using OAE-based techniques. Büki et al. [17] found significant phase changes (on the order of 20–30°), going from vertical position to − 30° head-down-tilt (HDT). Voss et al. [5] found large DPOAE level and phase changes between vertical and − 45° HDT positions, in the 0.5–4 kHz frequency range. Using Eq. (1), Avan et al. [6] estimated an ICP increase by about 6 mmHg (15°) in 1G going from seated to supine position, and a larger one (7, 12 mmHg, 19°, 34°) from seated to HDT position at tilt angles of − 6° and − 20°. In the pre-flight and post-flight sessions of an ICP spaceflight experiment [14], the sensitivity of the TEOAE test to changes of the body tilt angle was demonstrated in 1G, finding a systematic and significant progressive phase increase going from seated to supine (17°) and to − 15° HDT position (39°). In a recent ground experiment [15] on 15 normal-hearing subjects, an identical twin of the instrument used in the present study on the ISS was used to detect phase changes associated with postural changes, measuring an average phase change of 17° between the seated and supine positions, which would correspond, according to Eq. (1), to about 6 mmHg of ICP increase in the supine position.

Interestingly, three 1G body-tilt experiments [7, 15, 16] measuring the OAE (either DPOAE or TEOAE) phase yielded similar average phase difference between seated and supine conditions (16–17°), which according to Eq. (1), would correspond to about 6 mmHg of ICP increment in the supine position. The comparison with direct ICP measurements (see Table 1) suggests that, in postural experiments, the OAE-based techniques could underestimate the actual ICP changes.

### Parabolic Flight Experiments in Transitory Microgravity

In a parabolic flight experiment, direct ICP measurements [14] on hematological patients demonstrated, during free-falling (0G) sections, an ICP decrease by about 4 mmHg, with respect to the baseline 1G supine position, much lower than that measured going from seated to supine position in 1G (of order 11 mmHg). A steady state 0G condition (such as that of ISS astronauts) would therefore imply an ICP level intermediate between the seated and supine 1G levels (and closer to the supine one). They concluded that the SANS risk for astronauts could be associated with the prolonged absence of the restoring effect of the low-ICP condition that one experiences every day in 1G while standing or seated, for almost two-thirds of each day.

The OAE-based method for the ICP estimate was also used to evaluate the transitory effect of short-term exposure to microgravity during parabolic flight sections [7]. Using Eq. (1), an average increase of the ICP relative to the 1G seated baseline of 11 mmHg (a 30° phase increase was measured) was estimated. The same authors had found a smaller ICP increase (6 mmHg, 15°) going from seated to supine position in 1G.

Taken together, these two parabolic flight studies would suggest that the OAE-based technique could be more sensitive to transitory 0G than to postural changes and that full equivalence between OAE-based estimates and direct ICP measurements is not guaranteed.

### 0G Experiments on the ISS

In a recent comprehensive study [16], using a wide set of indirect ICP indicators, the phase of Transient Evoked Otoacoustic Emissions (TEOAE) was recorded from the right ear of 13 astronauts during their long term missions on the ISS. On average, during spaceflight, a significant average phase decrease below the pre-flight supine value (− 19.7° on flight day FD45), and no significant change (+ 2.4°, compatible with a null result) with respect to the pre-flight seated value were found. It may be worth mentioning that, for the only one subject with a diagnosis of optical disc edema, the authors reported instead a very large increase of TEOAE phase during spaceflight (see their Fig. 2).

## Study Description

The phase of the wave-fixed [18], zero-latency (ZL) component of the distortion product OAEs (DPOAEs), may be theoretically predicted to be a reliable otoacoustic quantity for detecting subtle ICP changes. Indeed, due to cochlear scaling symmetry, this phase is intrinsically almost frequency-independent, and its variation is almost univocally related to the variation of the middle ear impedance parameters, and therefore to the ICP change. Even in the apical range (below 1.5 kHz), where the DPOAE phase is a monotonically decreasing function of frequency, due to the cochlear scaling-symmetry breaking [19, 20], the DPOAE group delay is very small, of order 1–2 ms. This intrinsic feature of the ZL DPOAE phase allows one to average the phase differences over the whole frequency range without 2π ambiguity, which may not be granted otherwise, particularly in the presence of noise. The rapidly rotating phase of place-fixed DPOAE reflection component, as well as that of the TEOAEs, could be significantly affected also by changes of the gain and bandwidth of the cochlear amplifier [21], which cannot be ruled out in prolonged microgravity conditions, as also suggested by the SOAE sensitivity to postural changes [22]. Indeed, the increased intra-cochlear fluid pressure could negatively affect the performances of the cochlear amplifier, e.g., by changing its working point or directly affecting the OHCs, the relation between cochlear gain, bandwidth, and phase-gradient delay is well-established [23].

Acoustic Diagnostics [24] is an Italian Space Agency (ASI) experiment dedicated to monitoring the hearing function during long-term missions on the ISS. In the frame of its mission of promoting and fostering the culture of space, ASI provides access to the ISS as a laboratory in space. The payload was developed and tested for space with the technical assistance of ALTEC and used on board the ISS by five astronauts to perform DPOAE tests on themselves. Advanced data acquisition and analysis techniques and effective double-stage insulation (eartips + earmuffs) from ambient noise were designed to get high signal-to-noise ratio (SNR) measurements of complex DPOAE spectra despite the high environmental noise level of the ISS (of the order of 55–60 dBA in the Columbus section). DPOAE spectra were recorded pre-flight, in-flight, and post-flight, and the two DPOAE components (distortion and reflection) were unmixed using time–frequency analysis [25]. The change of the phase of the wave-fixed distortion component, averaged over the 1–4 kHz range, defined here as Φ _14_, provides an indirect monitor of the variation of the astronauts’ ICP during the mission, assuming the validity of a linear relation of the type of Eq. (1).

## Methods and Materials

Five astronauts (mean age 46 ± 3 years) were enrolled in the study. To preserve anonymity, the astronauts are not identified in this study, and the individual age, sex, and mission durations are not reported. The study protocol was approved by the Ethics Committee of the University of Roma La Sapienza, by the NASA and ESA Review Boards, and all astronauts gave their informed consent to their inclusion in the study.

High frequency-resolution (20 Hz) complex DPOAE spectra were recorded before, during, and after long term ISS missions (6–9 months). The DPOAE pre-flight baseline data collection (BDC) sessions were performed two to four months before launch in the seated position. Five to six recordings were taken on board the ISS at 1 to 3-month intervals. The first post-flight BDC was performed in the seated position within one and two weeks after return to Earth, and the second post-flight BDC was performed four to seven months later, to assess long-term ICP recovery. The BDC sessions were conducted either at the ESA European Astronaut Center (EAC, Cologne, Germany) or at the NASA Johnson’s Space Center (JSC, Houston, TX, USA), on a few occasions with the kind help of the JSC audiology specialist Dr. R. Danielson. Before each BDC DPOAE test, middle ear transmission was also assessed by tympanometry.

Probe positioning in the ear canal may be a critical issue, particularly when astronauts have to perform the measurements on themselves. In our case, the necessity of wearing earmuffs to isolate the microphone from ambient noise and the use of commercial rubber ear tips made correct probe orientation and fitting difficult for astronauts with a peculiar ear canal shape. Poor reproducibility and stability of the stimulus signals, observed adjustment of the probe in the ear canal by the astronaut during the recording session, insufficient SNR were the exclusion criteria that forced us to remove from the data set 5 DPOAE measurement out of a total of 82. Overall, 77 DPOAE recordings were included in the analysis (8 pre-flight, 50 in-flight, and 19 post-flight).

Two identical Acoustic Diagnostics systems (same hardware and software) were used for the DPOAE measurements on the ISS and for the BDC ground sessions. The DPOAE response was recorded at frequency *f*_DP_(*t*) = 2*f*_1_-*f*_2_ in the 1–6 kHz range, with high (20 Hz) frequency resolution, using two swept-tone (slow ascending linear chirps) stimuli *f*_1_(*t*) and *f*_2_(*t*). The speeds of the stimulus chirps were set in order to get an 800 Hz/s response chirp at the *f*_DP_ frequency. The stimuli were digitally generated and fed to the ER-2 loudspeakers (Etymotic Research) through a cDAQ NI9260 24-bit AO board, while the signal measured by the ER-10B + microphone (Etymotic Research) was preamplified and synchronously digitized by a NI9234 24-bit AI board (National Instruments), under the control of a custom Labview software (National Instruments). The response waveform was Fourier analyzed in N = 250, 50% overlapping, Hanning-windowed frames of 50-ms duration. This way, the spectral resolution of the Fourier analysis matches the variation of *f*_DP_ between consecutive frames, for optimal use of the acquisition time. Forward pressure calibration of the stimulus level (FPL) was performed before each measurement in the ear canal after probe insertion, and the DPOAE pressure level emitted by the eardrum (EPL) was computed, using in both cases the Thevenin calibration of the probe loudspeakers [26], which was performed before the launch of the payload, for both instruments. Using a differential method, no phase correction was applied, to avoid introducing differences with respect to the original studies that provided the conversion factor of Eq. (1). A total measurement time of about 3–5 min, dependent on the SNR, was necessary for testing each ear, corresponding to the acquisition of *N* = 30 synchronously averaged high-SNR frames for each frequency bin. For each chirp, a rejection criterion has been applied to remove noisy frequency bins. The noise rejection threshold was set based on the ambient noise level (typically about 55–60 dBA), measured before each session using the NASA EHS Acoustic Monitor. Time–frequency filtering [25] of the complex spectra was used to separate the distortion and reflection components, based on their different group delay, with a further SNR advantage of about 6–14 dB (increasing with frequency by 3 dB/oct).

Similarly to Sisto et al. [15], the average phase Φ_14_, calculated over the 1–4 kHz interval as the arithmetic mean of the unwrapped phase, was chosen as a stable observable parameter, and compared for each frequency to its “baseline” pre-flight value. Hence, this phase difference can be used as an indirect indicator of ICP changes. The linear relation of Eq. (1), established by Avan and coworkers, provides the conversion factor to convert the change in DPOAE phase to ICP change. Sisto et al. [15] used a slightly different frequency interval (1–3 kHz) for the detection of the effect of postural changes, because in that experiment the SNR in the low frequency range was higher than on the ISS.

### Statistical Analysis

The statistical significance of the observed phase changes was evaluated, using the software R (version 4.1.0, R Foundation for Statistical Computing, Vienna, Austria), by sorting the recordings in four groups: “baseline,” including the pre-flight BDC recordings, “return” and “2nd post-flight,” including, respectively, the first and second post-flight BDC recordings, and “in-flight,” including all ISS recordings. As the Φ_14_ data distribution did not pass a preliminary Shapiro–Wilk normality test, a Kruskal–Wallis test was used, which is the non-parametric analogue of a one-way ANOVA, to test the null hypothesis that the data originate from the same distribution. In the post-hoc analysis, a non-parametric pairwise Wilcoxon test was performed with the Bonferroni adjusted-*p*. A multivariate mixed-effect linear regression was also used. In this approach, particularly useful in case of non-independent measures performed on the same subject, the subject is considered as a random variable. The DPOAE phase variation was treated as a continuous numeric variable and the four levels factor “group” was introduced to distinguish among the four session groups. The factor “Ear”, distinguishing right and left ear was also included into the model. The statistical significance of the model itself was tested by fitting two models, one including the fixed effect factor of interest, i.e., “group,” and the other one not including it. The two models were compared by means of an ANOVA test. In this analysis, the “in-flight” group included all 50 ISS measurements, with no attempt to discriminate among different ISS sessions.

To follow the evolution of the average ICP variation during the ISS mission, and after return, the data from all astronauts were also sorted into five different groups: FD < 60, 60 < FD < 120, FD > 120, RD < 14, and 120 < RD < 210, where FD is the day of the in-flight measurement (relative the day of the launch *L*) and RD that of the post-flight measurement relative to the day of the return to Earth.

## Results

### Astronaut Data Collection

DPOAE complex spectra were recorded from both ears of five astronauts in the seated upright position, pre-flight (once, about 1–3 months before long term 6–9-month ISS missions), in-flight (four to six times) in a straight relaxed body position, and post-flight (twice, within two weeks from return and 4–7 months later). All subjects had normal hearing sensitivity, as evaluated by pure tone audiometry and tympanometry in the BDC sessions, and a well-measurable DPOAE response.

The baseline value of the phase was defined as that measured in seated position in the pre-flight baseline data collection (BDC) measurements. For one astronaut, pre-flight baseline measurements were missing, and the late 2nd post-flight measurements was used in that case as the baseline value, assuming full recovery to the pre-flight baseline condition after several months in 1G. Such an assumption is supported by what was observed in the other ears. Three out of ten post-flight measurements were taken in the supine position, due to a communication failure among the team. Five DPOAE recordings out of a total of 82 measurements had to be excluded from the analysis due to insufficient SNR and/or unstable probe placement, which was detected by monitoring the level and reproducibility of the stimuli.

### Effect of Body Posture in 1G

To compare our results to those of other studies using the supine position as 1G baseline and to test the sensitivity of our method, we have used the same instrument of the pre- and post-flight BDC sessions to evaluate on fifteen healthy subjects the typical average ZL DPOAE phase change that we measure in the same ear for different postures [21]. Using a post-hoc pairwise Wilcoxon test, we found a significant increase (*p* < 0.01) of order 15–20° immediately (within the time duration of a measurement session, of order 5 min) going from seated to supine, no significant change within a 10–15-min stabilization period, and full recovery immediately after returning to the seated position. In a further experiment, we are also checking the possible effect of different types of horizontal (supine vs. fetal lateral with straight neck) and (seated vs. standing) vertical body postures. Preliminary results show no significant difference between the seated and the standing position and between the supine and the fetal lateral position and confirm the size of the significant difference previously reported between horizontal and vertical postures.

### DPOAE Phase Changes in Prolonged ISS Microgravity

**Fig. 1:**
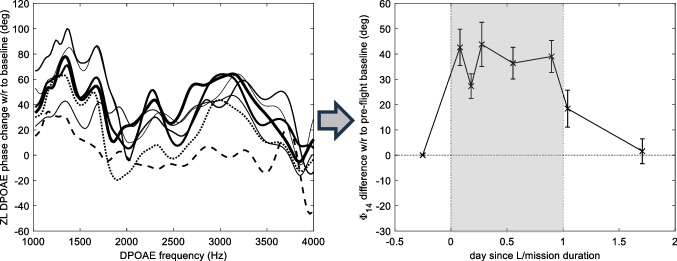
Left: high-resolution ZL DPOAE phase difference, with respect to pre-flight baseline phase, in the *f*_DP_ = 1–4 kHz range. Solid lines of thickness increasing with time identify the five in-flight sessions. The first and second post-flight BDCs are plotted as dotted and dashed lines, respectively. Right: time course of the Φ_14_ change during and after the ISS mission (shaded area)

The ZL DPOAE phase change with respect to the pre-flight seated baseline is reported as an example in Fig. 1 for the left ear of subject D, for seven measurement sessions, in the frequency range 1–4 kHz, where the SNR was generally highest for all subjects. As phase is intrinsically sensitive to noise, large random phase fluctuations were present in the original DPOAE spectra, which are significantly reduced by time–frequency filtering the ZL component. From the left panel of Fig. 1, one can get a first visual impression of the systematic increase of the DPOAE phase in microgravity. In this case, the ZL DPOAE phase increases significantly with respect to the pre-flight baseline value during the time spent on board the ISS (solid lines of thickness increasing with time), and recovers after return to Earth (dotted and dashed lines). The rather large residual fluctuations of the ZL DPOAE phase difference suggest the importance of considering as a more stable parameter the arithmetic mean Φ_14_ of the unwrapped phase change over the whole 1–4 kHz range. This can be unambiguously done, thanks to the slow intrinsic variation (much smaller than a cycle) of the wave-fixed ZL DPOAE component phase over the whole frequency interval. The result is shown in the right panel of Fig. 1, as a function of time spent on the ISS, normalized to the total mission duration to avoid indirect identification of the subject. The choice of the 1–4 kHz range is a compromise, justified by three facts: (a) the stability of the phase measurements increases with the width of the considered frequency band; (b) the sensitivity of the DPOAE phase to ICP was predicted theoretically [6] and verified experimentally [9] to be decreasing with frequency; and (c) the instrumental noise level decreases with frequency. We also checked “a posteriori” that the results do not change significantly if a 1–3 kHz range is used instead.

**Fig. 2:**
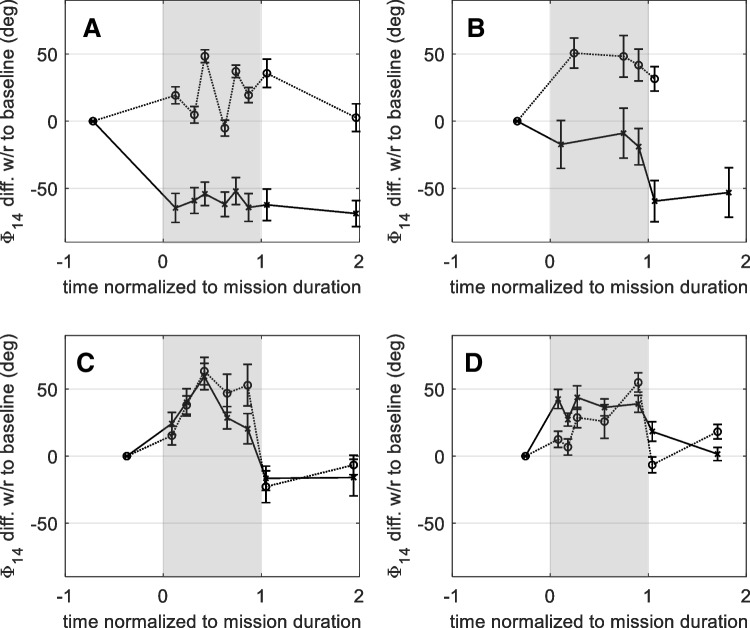
Φ_14_ change with respect to the seated baseline as a function of time relative to the launch day *L*, normalized to the mission duration, for the four subjects **A**, **B**, **C**, and **D**, with available pre-flight data. In subject E, with unavailable pre-flight data, the 2nd post-flight BDC phase was used as reference, and the individual data are not shown for privacy reasons

In Fig. 2, the Φ_14_ change with respect to the baseline phase is shown for the four subjects with available pre-flight data, as a function of the time spent on the ISS (normalized to the mission duration to preserve anonymity of the subjects). In two subjects (C and D), one can detect a Φ_14_ increase with respect to the baseline during the whole mission on the ISS, a recovery to the pre-flight phase after return to terrestrial gravity, and a good positive correlation (0.54 and 0.90, respectively) between the time courses measured in the two ears, with an Φ_14_ increase, averaged over all the in-flight data of each subject of 32° and 39°, respectively, which according to Eq. (1), would correspond to ICP increase of 12 and 14 mmHg. In the other three subjects, the average phase measured in-flight was systematically increased with respect to the 1G baseline only in one ear. The in-flight time course of the absolute phase was similar in the two ears, as visible in Fig. 2, but the difference with respect to the pre-flight baseline was systematically different, and negative in ears A left and B left. Although this observation, as well as that the post-flight phases are also systematically lower than the pre-flight baseline, suggests the possibility of a systematic error in these two pre-flight sessions, which obviously affects all the following phase differences, the data were included in the statistical analysis, because no evident sign of anomaly was noted in the calibration data. The only plausible reason for expecting a different relation between DPOAE phase and ICP in the two ears of the same subject would be a strongly asymmetric patency of the cochlear aqueduct, which is not very likely to occur in healthy subjects of this age. Therefore, this lack of agreement between the two ears of the same subject should be considered as hint for the occurrence of systematic errors in the baseline measurements, which could be overcome by repeating the baseline measurement in different pre-flight sessions, possibly in different postures, and by achieving a more reproducible probe positioning in the ear canal.

**Fig. 3:**
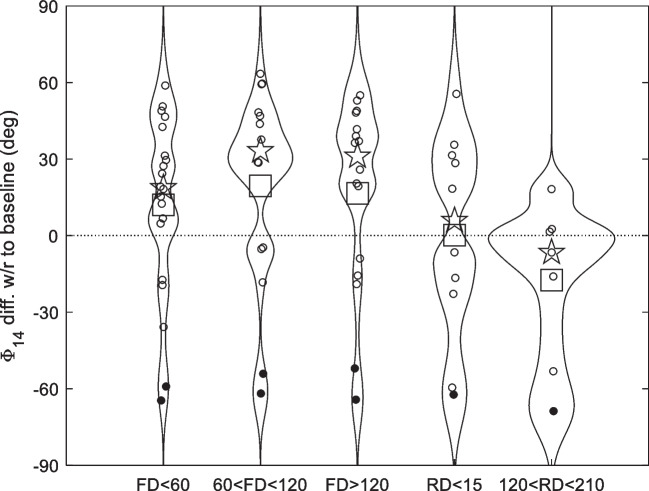
Violin plots summarizing the Φ_14_ change, with respect to the baseline value, in five-time intervals during and after the ISS mission. The 50 in-flight data are sorted in three-time intervals within the mission duration, the first sixty flight days (FD) after the day of launch, the next sixty days, and the days on the ISS after FD120, including, respectively, 20, 14, and 16 measurements. The first post-flight measurement was taken within the second week after the day of return to Earth, and the second post-flight measurement 4–7 months after that. The day of the post-flight measurement RD is measured from the day of the return to Earth. As the distribution is strongly non-gaussian, the median (pentagrams) is also shown along with the average (squares), although none is representative of the distributions. The points from ear A left are marked with a filled black circle

In Fig. 3, the estimated change of Φ_14_ with respect to the baseline is represented by violin plots, grouping the data of all ears within five-time intervals, to follow, to some extent, the temporal evolution of Φ_14_ during the ISS mission and after return to Earth. During the permanence on board the ISS, the average and median Φ_14_ increase with respect to the seated baseline level, up to about 20° and 30°, respectively, and recover to the baseline value after return to Earth. This overall behavior agrees qualitatively with what was generally observed also in most of the individual data (in seven out of ten ears), with large fluctuations and significant variability of the max phase increase (of order 50° in seven ears).

### Statistical Considerations

**Fig. 4:**
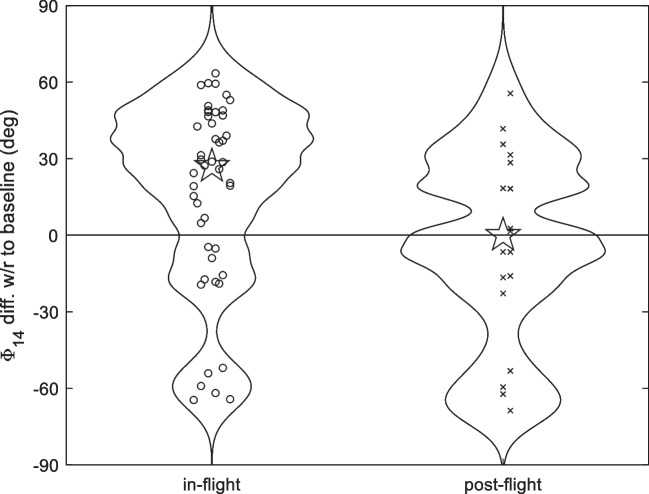
Violin and swarm plots summarizing the distribution of the Φ_14_ change *w*/*r* to the baseline, in the other BDC 1G sessions (crosses) and in spaceflight 0G (circles) groups, for all astronauts. The medians of the two distributions are indicated by a pentagram

As anticipated in the Methods Section, the distributions of both the post-flight and in-flight Φ_14_ differences from baseline are both non-gaussian, as shown by Fig. 2 and, particularly, by Figs. 3 and 4, where violin plots are combined with the swarm plot of the individual data. Therefore, average values are not representative of the whole data set behavior, while the median (shown by a pentagram) may be a slightly more reliable indicator. Note also that all the six strongly negative differences in the lower part of the violin “in flight” plots of Figs. 3 and 4 come from the A left ear.

The Kruskal–Wallis by ranks test yielded *p* = 0.0006 for the significance of the factor “group” in explaining the phase distributions. The post-hoc analysis showed a significant difference only between the “in-flight” and the “pre-flight” conditions according to the pairwise Wilcoxon test (adjusted *p* = 0.0003). The mixed-effect analysis demonstrated the significance of the variable “group,” *p* = 0.0006. A Φ_14_ increase was observed on the ISS, with an average difference of 16 ± 5° (*p* = 0.001) with respect to the seated baseline. Note that, although the actual size of the ICP increase depends on the validity of a linear relation such as Eq. (1), and on the precision of its numerical coefficient, the significance of the Φ_14_ increase on the ISS, as indirect indicator of ICP, is independent of its validity.

## Discussion

### Average and Median Phase Changes

A statistically significant Φ_14_ increase, with respect to the seated baseline (average 16°, median 27°, in some ears as large as 60°) occurs during the astronauts’ permanence in microgravity on board the ISS, with respect to the seated baseline. If the relationship of Eq. (1) holds between Φ_14_ and ICP, the average ICP changes estimated during spaceflight with respect to the seated position would be of about 6 mmHg, the median change would correspond to 10 mmHg and the largest observed changes to about 20–25 mmHg. This systematic behavior was observed in a large fraction of the examined ears (seven out of ten ears, with large inter-subject variability).

The Φ_14_ increase in 0G during spaceflight reported in the present study is relative to a baseline 1G seated posture, in which ICP is significantly lower than in the supine position. The same instrument used in the present study measured, in a ground experiment [15] on 15 volunteers, an average Φ_13_ (in that case the optimal SNR range was 1–3 kHz) increase of 17° between DPOAE measurements taken in the seated and supine position, corresponding, according to Eq. (1), to an ICP increase of 6 mmHg. These numbers could be used to compare our estimates of the ICP increase in 0G to those of the studies that use the supine position as a baseline.

Considering the large inter-subject variability and the small number of subjects, these average results are not inconsistent with the two previously mentioned parabolic flight experiments. One could note that Avan et al. [7] estimated a larger difference (+ 11 mmHg) between the 0G and 1G seated conditions than between the 1G supine and 1G seated conditions (+ 6 mmHg), while Lawley et al. [14] measured smaller ICP difference (+ 7 mmHg) between the 0G and 1G seated conditions than between the 1G supine and 1G seated conditions (+ 11 mmHg).

Our average results are not in agreement with the only other ISS 0G experiment [11], in which, using the TEOAE phase, a significant phase decrease (− 19.7°) was measured with respect to supine position in 1G, and no significant phase increment (+ 2.4°, consistent with zero) was measured in ISS 0G with respect to seated position in 1G. The average OAE phase and ICP changes in microgravity reported by the different experiments are summarized in Table 2.

**Table 2:** Microgravity experiments

Avan [7]	40 subjects	+ 30° (+ 11 mmHg) (parabolic 0G–seated 1G) − 32° (+ 11.8 mmHg) (parabolic 1.8G–seated 1G)
Lawley [14]	8 hematological patients	− 3.8 ± 2.9 mmHg (Parabolic 0G–supine 1G)
Jasien [16]	Right ear of 13 ISS astronauts	(− 19.7 ± 9)° (ISS 0G FD45–supine 1G) (+ 2.4 ± 9)° (ISS 0G FD45–seated 1G)
This study (2024)	Both ears of 5 ISS astronauts	+ 12° (ISS 0G FD < 60–seated 1G) + 19° (ISS 0G 60 < FD < 120–seated 1G) + 16° (ISS 0G FD > 120–seated 1G)

### Individual Phase Changes

One must consider that average values are fully representative for large data sets with Gaussian-like distributions only. The small number of subjects of the present study, the non-Gaussianity of the strongly bimodal distribution of the differential data imply that any quantitative average estimate is too sensitive to outliers (the median being a slightly more stable quantity) and may easily depend on the accidental inclusion/exclusion in the study of a single ear with inaccurate pre-flight baseline measurements.

On the other hand, individual data may be validated by comparing the results obtained independently for the two ears of the same subject. For two astronauts, a strong correlation was observed between the Φ_14_ time course of the two ears, which were measured independently, inserting the same probe first in one ear, and then in the other one. This correlation may be considered as a quality index of the data and suggests that the observed phase changes are likely related to some condition affecting similarly both ears during the whole mission (likely, but not necessarily, the ICP increase). In these two subjects, the average in-flight Φ_14_ change was 32° and 39°, respectively, which would correspond, using Eq. (1), to about 12 and 14 mmHg of ICP increase, respectively, significantly exceeding the typical ICP increase observed with our instrument between seated and supine 1G conditions [15], and in better agreement with the parabolic flight results of Avan et al. [6]. We note that the other OAE study performed on the ISS [16] reports, for the only subject with a clear diagnosis of optical edema, a very large TEOAE phase increase in the ISS 0G condition, relative to the seated, supine and HDT 1G conditions. Unfortunately, in that study, the OAE response is reported for one ear only of each subject.

### Study Limitations and Future Directions

As the calibration law of Eq. (1) was never tested directly using direct ICP measurements for our instrument and method, the significance of the Φ_14_ excess measured in-flight provides indirect evidence for an ICP increase with respect to the seated 1G condition, but it cannot provide an accurate estimate of its amplitude. The other main limitations of the present study are associated with the small number of subjects, and the uncertainties associated with the probe placement in the ear canal. For a fully meaningful comparison with similar studies, pre-flight baseline measurements both in the seated and in the supine position would have been necessary. A delicate issue is obtaining a reproducible baseline value, which would have required repeating the test in a second pre-flight BDC (e.g., after a few weeks) to evaluate the test–retest uncertainty. All this was not possible due to the limitations of the available crew time, because a large fraction of the BDC time was dedicated, according to the Acoustic Diagnostics protocol, to a full set of audiological measurements. The unstable probe placement could be overcome in a future experiment by using personalized earplugs modeled on the actual shape of each ear canal, particularly important in the case of irregular ear canal shapes.

A new ASI experiment (HESIOD) dedicated to the OAE-based ICP measure on the ISS is planned for the near future. With respect to Acoustic Diagnostics, the new payload will be optimized for the monitor of the DPOAE phase in the most sensitive frequency region. Indeed, the DPOAE optimal frequency range for ICP estimates has to be evaluated based on a trade-off between reproducibility of the measured phase, which is best in the frequency range in which the phase is independent of frequency and the SNR is highest (2–4 kHz), and the sensitivity of the measured phase to ICP changes, which seems to be maximal around 1 kHz [9].

A calibration of our method and instrument involving comparisons with direct ICP measurements is planned to specify the absolute size, and therefore the clinical meaning, of the ICP increase associated with the Φ_14_ changes.

## Conclusions

The measurement of average ZL DPOAE phase (Φ_14_) is proposed in this study as an objective, fast, and non-invasive indirect indicator of ICP increase in microgravity. We provide evidence for a significant increase of this indicator under prolonged microgravity conditions, with respect to the seated 1G baseline, in a sample of five astronauts during their missions on the ISS, with large inter-subject variability. The average ICP increase estimated in ISS 0G conditions is of the same order as that estimated with the same instrument going from the seated to supine position in 1G studies, but it is much larger than that for some specific subjects. In any case, one must consider [14] that an ICP increase of the order of (or smaller even than) that associated with taking the supine position in 1G during each nightly sleep, could have adverse effects on the astronauts’ vision, because it would be continuous on a time scale of several months.

## Data Availability

The codes used for the analysis will be made available on request. The access to sensitive raw data of the astronauts is restricted, by agreements made with ASI, ESA, and NASA, to the Acoustic Diagnostics PI and his research team only; therefore, these data will be supplied on request and after specific approval from the involved space agencies, omitting any details that could permit direct or indirect identification of the subjects, e.g., the duration of the ISS mission, age, and sex.

## Acronyms

ICP: Intracranial pressure
SANS: Spaceflight associated neuro-ocular syndrome
VIIP: Visual impairment and intracranial pressure
LP: Lumbar puncture
ISS: International space station
FD: Flight day
OAE: Otoacoustic emission
DPOAE: Distortion product OAE
TEOAE: Transient-evoked OAE
SOAE: Spontaneous OAE
ZL: Zero-latency
HDT: Head-down tilt
OHC: Outer hair cell
BDC: Baseline data collection
FPL: Forward pressure level
EPL: Emitted pressure level
